# Differential Gene Expression Associated with Altered Isoflavone and Fatty Acid Contents in Soybean Mutant Diversity Pool

**DOI:** 10.3390/plants10061037

**Published:** 2021-05-21

**Authors:** Dong-Gun Kim, Jae-Il Lyu, You-Jin Lim, Jung-Min Kim, Nguyen-Ngoc Hung, Seok-Hyun Eom, Sang-Hoon Kim, Jin-Baek Kim, Chang-Hyu Bae, Soon-Jae Kwon

**Affiliations:** 1 Advanced Radiation Technology Institute, Korea Atomic Energy Research Institute, Jeongup 56212, Korea; dgkim@kaeri.re.kr (D.-G.K.); jaeil@kaeri.re.kr (J.-I.L.); jmkim0803@kaeri.re.kr (J.-M.K.); nguyenhung@kaeri.re.kr (N.-N.H.); shkim80@kaeri.re.kr (S.-H.K.); jbkim74@kaeri.re.kr (J.-B.K.); 2 Department of Horticultural Biotechnology, Institute of Life Sciences & Resources, Kyung Hee University, Yongin 17104, Korea; yujin0213@khu.ac.kr (Y.-J.L.); se43@khu.ac.kr (S.-H.E.); 3 Department of Life Resources, Graduate School, Sunchon National University, Suncheon 57922, Korea

**Keywords:** soybean, isoflavone, oleic acid, seed development, gene expression

## Abstract

Soybean seeds are consumed worldwide owing to their nutritional value and health benefits. In this study we investigated the metabolic properties of 208 soybean mutant diversity pool (MDP) lines by measuring the isoflavone and fatty acid contents of the seed. The total isoflavone content (TIC) ranged from 0.88 mg/g to 7.12 mg/g and averaged 3.08 mg/g. The proportion of oleic acid among total fatty acids (TFA) ranged from 0.38% to 24.66% and averaged 11.02%. Based on the TIC and TFA among the 208 MDP lines, we selected six lines with altered isoflavone content and six lines with altered oleic acid content compared with those of the corresponding wild-types for measuring gene expression. Each of twelve genes from the isoflavone and fatty acid biosynthesis pathways were analyzed at three different seed developmental stages. Isoflavone biosynthetic genes, including *CHI1A*, *IFS1*, and *IFS2*, showed differences in stages and expression patterns among individuals and wild-types, whereas *MaT7* showed consistently higher expression levels in three mutants with increased isoflavone content at stage 1. Expression patterns of the 12 fatty acid biosynthetic genes were classifiable into two groups that reflected the developmental stages of the seeds. The results will be useful for functional analysis of the regulatory genes involved in the isoflavone and fatty acid biosynthetic pathways in soybean.

## 1. Introduction

Soybean is an important oilseed crop and is among the most widely used and healthy edible oils. Soybean oil is low in saturated fat, rich in essential fatty acids, an excellent source of vitamin E, and contains no cholesterol. The oil quality is primarily a function of the fatty acid composition. The ratio and amount of saturated and unsaturated fatty acids determine the physical, chemical, and nutritional values of the oil [1]. The average fatty acid composition of commercial soybean oil is approximately 12% palmitic (16:0), 4% stearic (18:0), 23% oleic (18:1), 53% linoleic (18:2), and 8% linolenic (18:3) acids [2]. These are the predominant fatty acids in soybean oil, but the ideal fatty acid composition depends on the specific use for which it is intended. Thus, modification of the fatty acid composition of soybean oil is beneficial for food and industrial applications.

Soybean plant growth and development are traditionally classified into six vegetative and eight reproductive (R) stages. R1 and R2 are stages of flowering, R3 and R4 encompass pod formation, and seed development occurs in stages R5–R8 [3,4]. The final seed composition of secondary metabolites, including isoflavones, is strongly influenced by environmental stresses during stages R5–R7 [5,6,7]. Seed development in stage R5 is characterized by a rapid increase in weight and nutrient accumulation, which continues until R6 [3]. Seeds in the R6 stage fill the pod cavity but are still immature [4]. Typical of the R7 stage, the seed coat color begins to change from green to either tan or yellow, depending on the cultivar [8]. At this stage, accumulation of dry weight ceases and the seed has attained physiological maturity [9]. Isoflavones accumulate in the seeds during the advanced stages of seed maturation [10,11], and isoflavone contents are strongly influenced by water availability during this period.

Isoflavones are polyphenolic secondary plant metabolites found in seedlings, flowers, and roots, and are especially abundant in seeds and leaves of soybean. Within seeds, diverse tissues have the ability to synthesize isoflavones [12]. Multigenic responses to abiotic stimuli influence soybean development and are highly variable throughout the plant, and among organs and tissues, with respect to the environment [10,13]. Genistein, daidzein, and glycitein, the known soybean isoflavones, are synthesized by a branch of the phenylpropanoid pathway. This extended metabolic route is also involved in the synthesis of other important compounds in plants, such as tannins, lignins, lignans, anthocyanins, flavones, flavonols, and the soybean phytoalexins, glyceollins, which are pterocarpans that possess antimicrobial activities [14]. The precursor in the pathway is the amino acid L-phenylalanine, which in the initial step is stripped of its amine group to produce cinnamic acid catalyzed by phenylalanine ammonia lyase (PAL). In the second and third reactions, cinnamate 4-hydroxylase (C4H) and 4-coumarate CoA ligase (4CL) convert cinnamic acid into *p*-coumaryol CoA. The first critical enzyme required for flavonoid synthesis is chalcone synthase (CHS), which is a multigene family in soybean, although not all copies are expressed in seeds at detectable levels. Other important enzymes in the pathway for isoflavone synthesis are chalcone isomerase (CHI), which converts chalcones to flavanones, and chalcone reductase (CHR), which is required for daidzein and glycitein formation. However, the enzyme that specifically differentiates isoflavone-producing plant species from those with no isoflavone content is isoflavone synthase (IFS), an endoplasmic reticulum (ER)-associated cytochrome P450 monooxygenase, that catalyzes 2,3-aryl ring migration of flavanones to their corresponding isoflavones [15,16,17]. In the soybean genome, IFS is present in two copies, IFS1 and IFS2 that differ by several amino acids. Both enzymes convert naringenin and liquiritigenin to genistein and daidzein, respectively. Despite their homology, IFS1 and IFS2 are differentially regulated at the transcriptional level. For instance, although both proteins contribute to the isoflavone content in the seed [18], expression of *IFS2* increases at advanced stages of seed development, whereas *IFS1* transcription remains relatively constant [10,12]. Moreover, only *IFS2* is induced in soybean hypocotyls and transgenic roots in response to pathogen attack [19]. 

Three types of fatty acid metabolic enzymes, namely stearoyl-acyl carrier protein-desaturases (encoded by *GmSACPD* genes), omega-6 desaturases (*GmFAD2* genes), and omega-3 desaturases (*GmFAD3* genes), largely determine the relative degree of unsaturated fatty acids and the contents of the C_18_ fatty acids stearate (18:0), oleate (18:1), linoleate (18:2), and linolenate (18:3) in vegetative and seed lipids. The Δ^9^ stearoyl-acyl carrier protein-desaturases are soluble enzymes localized to the stroma fraction of plastids that insert the first double bond to stearoyl-ACP (18:0-ACP) to produce oleoyl (18:1Δ^9^)-ACP. Thus, Δ^9^-stearoyl-ACP-desaturases perform a crucial step in C_18_ fatty acid biosynthesis because perturbation of *SACPD* expression and/or enzyme activity may modulate the relative cellular content of both stearate and oleate. Three alleles of *SACPD* have been identified and characterized from soybean. The soybean ω-6 oleate fatty acid desaturases (*FAD2* genes) are microsomal enzymes that initiate the primary route of polyunsaturated lipid biosynthesis by catalyzing the first extraplastidal desaturation to convert 18:1 esterified fatty acids to phosphatidylcholine to α-18:2 fatty acids [20]. Four ω-6 desaturase genes comprise the soybean *FAD2* gene family [21], consisting of *GmFAD2-1* and *GmFAD2-2* and their alleles [20,22,23], *GmFAD2-3* [24], and *GmFAD6* [20,22].

Radiation breeding is an effective method of inducing mutations in seeds and other propagative materials, such as pollen, whole plants, or tissue-cultured calli [25,26], and directly produces mutant varieties [27]. With regard to agronomic traits, gamma radiation frequently alters flowering, maturation traits, seed coat color, chloroplast number, and biomass yield in soybean [28,29]. Gamma radiation directly produces mutant varieties without the need to go through the otherwise lengthy and laborious process of conventional breeding. For that reason, mutation breeding has been used in various crops and ornamental plants, and has proven to be a promising means of producing new genetic variants [30,31].

Previously, we successfully constructed a soybean mutant diversity pool (MDP), which exhibited dramatic changes in agronomic and morphological traits [30]. In the present study, gamma-irradiated mutants were selected by screening for changes in isoflavone and fatty acid content using MDP lines. In addition, the relative expression level of genes associated with isoflavone and fatty acid biosynthesis was evaluated.

## 2. Results

### 2.1. Seed Isoflavone Content of 208 Soybean MDP Lines

**Figure 1:**
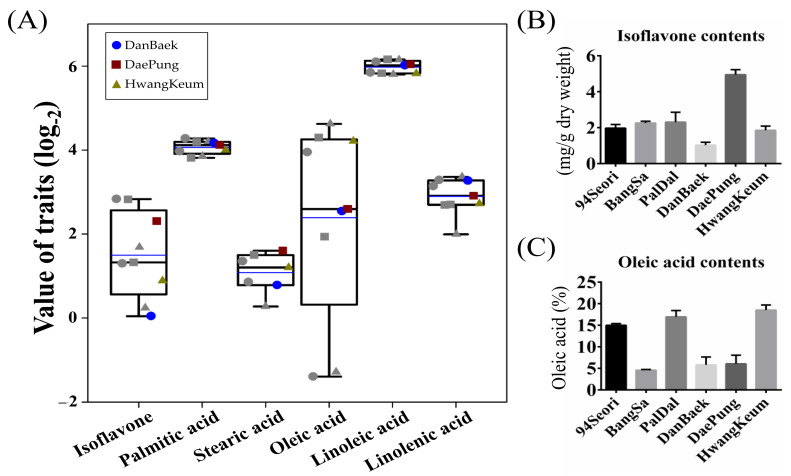
Changes in phytochemical traits (isoflavone and fatty acid contents) of 15 selected MDP lines compared with six Korean cultivars of soybean. (**A**) Box plots of the phenotypic distributions for three wild-type cultivars and their selected MDP lines (six isoflavone-altered lines comprising DB-088, DB-064, DP-084, DP-093, HK-17, and HK25-165; and six oleic acid-altered lines comprising DB-075, DB-041, DP-056, DP-184, HK-30, and HK-37; represented by gray symbols). The data are presented as log_2_-based mean values for individual lines. Distribution of the phytochemical traits among the six Korean cultivars is presented for (**B**) isoflavone (mg/g dry weight) and (**C**) oleic acid (%) contents.

First, we assessed the total isoflavone content (TIC) in seeds of the 208 MDP lines. The TIC ranged from 0.88 mg/g (KAS360-22) to 7.12 mg/g (DB-088) with an average of 3.08 mg/g for the 208 MDP lines. With regard to TIC, the seven wild-type cultivars were ranked, in descending order, as DP (4.95 mg/g), PD (2.31 mg/g), BS (2.26 mg/g), 94Seori (1.97 mg/g), HK (1.85 mg/g), DB (1.03 mg/g), and KAS360-22 (0.88 mg/g) (Supplementary Table S1, Figure 1B). For the 60 DP-mutants and 64 DB-mutants, the TIC ranged from 1.47 to 7.08 mg/g and from 1.20 to 7.12 mg/g, respectively. The TIC for the three mutant populations derived from PD, BS, and HK were relatively lower and ranged from 1.14 to 4.07 mg/g, 1.05 to 1.51 mg/g, and 1.08 to 3.21 mg/g, respectively. Detailed information on the isoflavone contents for each mutant-population is summarized in Table 1. We selected nine MDP lines that exhibited either increased isoflavone content (IIC: DB-088, 7.12 mg/g; DP-084, 7.08 mg/g; and HK-17, 3.21 mg/g) or decreased isoflavone content (DIC: DB-064, 1.34 mg/g; DP-093, 2.51 mg/g; and HK25-165, 1.18 mg/g), and the corresponding wild-type cultivars (DB, DP, and HK) for gene expression analysis. Also, variations of the isoflavone content and fatty acid content levels of the selected mutants are shown in Figure 1A.

### 2.2. Seed Fatty Acid Content of 208 Soybean Lines

For investigation of the fatty acid composition of the seeds, we measured the concentrations of five saturated/unsaturated fatty acids, comprising palmitic (16:0), stearic (18:0), oleic (18:1), linoleic (18:2), and linolenic (18:3) acids, by GC-MS analysis. The proportion of palmitic acid ranged from 12.42% to 21% of the total fatty acid (TFA) composition in the 208 MDP lines (Supplementary Table S2). Interestingly, KAS360-22 showed a comparatively high percentage stearic acid content (22.99%), which was approximately 11-fold higher than the average percentage for the 208 MDP lines (2.08%). However, the KAS360-22-W mutant derived from KAS360-22 exhibited a similar percentage (3.66%) to that of other mutant lines. Linoleic acid, a major fatty acid compound in soybean seeds, constituted more than 50% of the TFA composition in the 208 MDP lines. The proportion of oleic acid in the 208 MDP lines ranged from 0.38% to 15.43% in the DB-mutant population, 1.54% to 19.83% in the DP-mutant population, and 0.41% to 24.66% in the HK-mutant population (Table 2). With regard to the percentage oleic acid, the wild-type cultivars were ranked, in descending order, as HK (18.52%), PD (16.95%), 94Seori (14.98%), DP (6.05%), DB (5.83%), BS (4.57%), and KAS360-22 (2.25%) (Figure 1C). The proportion of oleic acid differed significantly among the 208 MDP lines and thus represents variation useful for genetic engineering. Therefore, for further genetic analyses, we focused on the change in oleic acid content among the MDP lines. After screening the oleic acid content among the 208 MDP lines, we selected six mutants that exhibited either increased oleic acid proportions (IOC: DB-075, 15.43%; DP-056, 19.68%; and HK-30, 24.66%) or decreased oleic acid proportions (DOC: DB-041, 0.38%; DP-184, 3.81%; and HK-37, 0.41%), and the corresponding wild-type cultivars (DB, DP, and HK).

### 2.3. Differential Expression of Isoflavone Biosynthesis Genes during Seed Development

**Figure 2:**
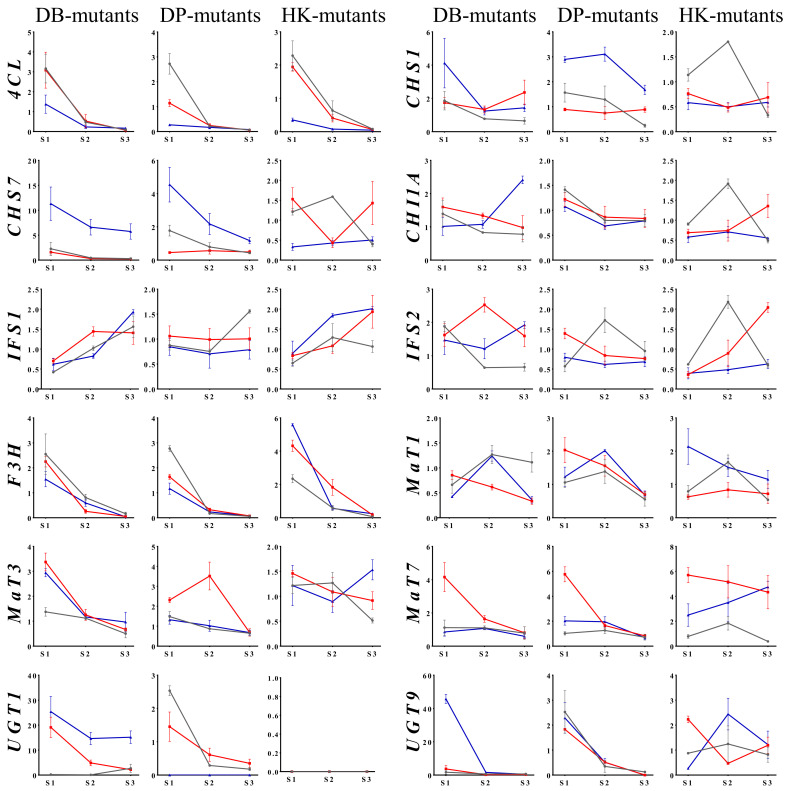
Relative expression level of isoflavone biosynthesis genes in seeds of soybean ‘Danbaek-mutants’ (Black line, DB; red line, DB-088; blue line, DB-064), ‘Daepung-mutants’ (Black line, DP; red line, DP-084; blue line, DB-093), and ‘Hwangkeum-mutants’ (Black line, HK; red line, HK-17; blue line, HK25-165) MDP lines at three developmental stages. Expression levels were estimated using quantitative real-time PCR analysis. The relative expression level was normalized to the internal reference gene *ELF1B*.

In the present study, we analyzed the expression patterns of 12 genes involved in isoflavone biosynthesis in soybean using qRT-PCR (Figure 2). The IIC (DB-088, DP-084, and HK-17) and DIC mutants (DB-064, DP-093, and HK25-165), and the corresponding wild-type cultivars (DB, DP, and HK) were selected for analysis of the differential expression of isoflavone biosynthetic genes at seed developmental stages 1, 2, and 3. The expression level of upstream genes in the flavanone biosynthesis pathway, comprising three genes acting upstream of *CHI* (*4CL*, *CHS1*, and *CHS7*), *CHI1A*, and two genes leading to the synthesis of isoflavones or flavonols from flavanone (*IFS1* and *IFS2*, *F3H*, *MaT1*, *MaT3*, *MaT7*, *UGT1*, and *UGT9*), was evaluated in the nine selected lines. The *4CL* gene showed significant differences in expression level between IIC and DIC lines, which was consistently higher at stage 1 in the DB-, DP-, and HK-mutants. *CHS1* and *CHS7* exhibited similar expression patterns between the DB- and DP-mutants, whereas the HK-mutants showed a different expression pattern compared with those of the DB- and DP-mutants. *CHI1A*, *IFS1*, and *IFS2* showed differences in stages and expression pattern depending on the individual or wild-type cultivar. For example, the IIC mutant lines DB-088, DP-084, and HK-17 showed differential expression of *IFS2* at stage 2, stage 1, and stage 3, respectively, based on the maximum relative expression values. These results indicated that *IFS2* was involved in isoflavone accumulation, but the regulation timing differed owing to the genetic background. In contrast, *MaT7*, an isoflavone-specific malonyltransferase, consistently showed higher relative expression levels at stage 1 in the three IIC lines, and thus may be the main contributor to isoflavone biosynthesis in soybean seeds. Interestingly, *UGT1* expression was not detected in HK-mutants and HK.

### 2.4. Differential Expression of Fatty Acid Biosynthesis Gene during Seed Development

**Figure 3:**
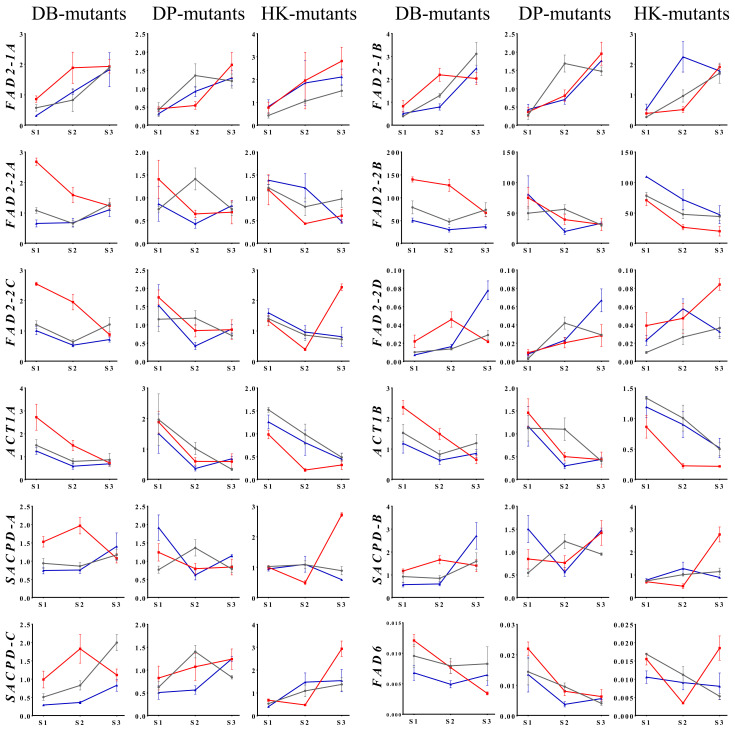
Relative expression level of fatty acid biosynthesis genes in seeds of soybean ‘Danbaek-mutants’ (Black line, DB; red line, DB-075; blue line, DB-041), ‘Daepung-mutants’ (Black line, DP; red line, DP-056; blue line, DB-184), and ‘Hwangkeum-mutants’ (Black line, HK; red line, HK-30; blue line, HK-37) MDP lines at three developmental stages. Expression levels were estimated using quantitative real-time PCR analysis. The relative expression level was normalized to the internal reference gene *ELF1B*.

Based on the oleic acid content in seeds of the MDP lines, we analyzed the differential expression of fatty acid biosynthetic genes at three seed developmental stages. The qRT-PCR analyses were conducted with primers that targeted genes up- or downstream of oleic acid (18:1) biosynthesis (Figure 3). To confirm the interaction between oleic acid content and related gene expression, the expression of 12 major structural genes, including *FAD2-1A*, *FAD2-1B*, *FAD2-2A*, *FAD2-2B*, *FAD2-2C*, *FAD2-2D*, *SACPD-A*, *SACPD-B*, *SACPD-C*, *ACT1A*, *ACT1B*, and *FAD6*, was analyzed at three seed developmental stages for nine MDP lines. The IOC mutant DB-075 showed consistently higher expression levels of expression stage (ES) I genes than those of DB and DB-041. In contrast, ES III genes showed a similar or decreased expression level in DB-075 compared with those of DB and DB-041 at stage 3. At stage 2, DB-075 showed consistently high expression levels for 11 major structural genes, except *FAD6*, compared with those of DB and DB-041. Expression patterns differed somewhat between the IOC mutant DP-056 and the DOC mutant DP-184; for example, *FAD2-2C* and *SACPD-C* expression differed significantly at stage 2, but no significant differences between DP-056 and DP-184 were observed for other genes analyzed. The HK-30 mutant showed higher relative expression levels of ES III genes compared with those of HK and HK-37; these expression patterns differed from those observed for the DB- and DP-mutants.

## 3. Discussion

In this study, we investigated the metabolic properties, represented by the contents of isoflavones and five fatty acids, of the seeds of 208 MDP lines. The TIC ranged from 0.88 mg/g (KAS360-22) to 7.12 mg/g (DB-088) and averaged 3.08 mg/g. The TIC of the seven wild-type cultivars were ranked, in descending order, as DP (4.95 mg/g), PD (2.31 mg/g), BS (2.26 mg/g), 94Seori (1.97 mg/g), HK (1.85 mg/g), DB (1.03 mg/g), and KAS360-22 (0.88 mg/g) (Table 1, Supplementary Table S1). A previous study reported that, among five Korean soybean cultivars analyzed, ‘Saegeum’ showed the highest TIC (1.22 mg/g) in the seeds [32]. This content is similar to that of DB observed in the present study and was 5.9-times lower than that of the DB-088 line. These results indicate that the DB-088 line shows the highest seed TIC reported for the Korean region. Park et al. [33] reported that seeds of the cultivar ‘Bosek’ showed the highest TIC (3.43 mg/g) among 106 Korean soybean cultivars surveyed, but this content was lower than that of the DB-088 line. In addition, Choi et al. [34] reported a TIC that ranged from 1.827 to 5.777 mg/g in 49 Korean soybean accessions, and Azam et al. [35] reported a TIC that ranged from 0.745 to 5.253 mg/g in 1168 China soybean accessions. These results confirmed that the TIC of DB-088 was approximately 1.2- to 9.5-times higher than that of other accessions. 

Soybean oil with high oleic acid content has the potential to improve the overall oil composition profile because oleic acid is an important component of soybean oil. Therefore, many researchers have attempted to develop soybeans with the desired fatty acid phenotypes through breeding [36]. In the present study, we measured the contents in the seed of five saturated/unsaturated fatty acids, consisting of palmitic (16:0), stearic (18:0), oleic (18:1), linoleic (18:2), and linolenic (18:3) acids, by GC-MS analysis. The proportion of oleic acid in 208 MDP lines ranged from 0.38% to 15.43% in the DB-mutant population, 1.54% to 19.83% in the DP-mutant population, and 0.41% to 24.66% in the HK-mutant population. The oleic acid content ranged from 0.38% to 24.66% and averaged 11.02% (Table 2, Supplementary Table S2). Our MDP lines identified a variety of oleic acids. Therefore, it was confirmed that the oleic acid of the mutant lines increased in a range from approximately 1.3 to 5.3-times compared to the wild-type. In previous studies, Rahman et al. [37] conducted X-ray irradiation of soybean seeds and selected the M23 line, which showed an approximately two-fold increase in oleic acid content in the seed compared with that of the wild-type. This mutant line showed 46.1% oleic acid content in the seed, but also exhibited a parallel decrease in linoleic acid content. In another study, the increase in oleic acid content of the transgenic varieties with FAD-2 downregulation was approximately 4-times higher than that of soybean wild-type [38]. As an extreme case, Lee et al. [39] developed the cultivar ‘Hosim’ from the cross 17D × S08-14788, which shows a high proportion of oleic acid (79%) in the seed. These results confirmed that the MDP lines were approximately 3.2 to 207-times lower than that of ‘Hosim’ cultivar. In addition, Choung [40] analyzed the fatty acid composition of 563 soybean accessions and reported the proportions 13.3–14.3% oleic acid, 38.2–61.5% linoleic acid, and 5.0–11.5% linolenic acid. Similarly, Song et al. [41] reported the oleic acid content of 379 soybean accessions (128 Korean, 214 Chinese, and 37 USA accessions), which ranged from 15.3% to 56.0% (mean 28.2%). Commodity soybean oil typically contains 20–25% monounsaturated oleic acid but recently lines producing higher proportions of oleic acid have entered commercial production [33]. A variety of genetic and biotechnological approaches to increase the oleic acid content in soybean seeds are currently being explored [42].

**Figure 4:**
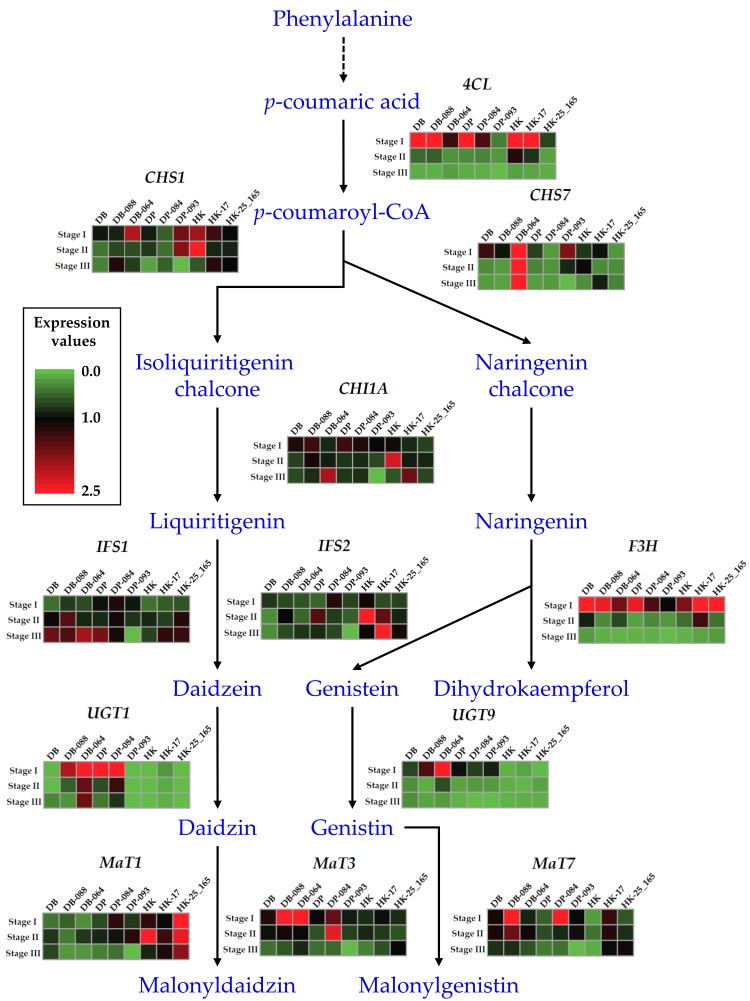
Transcript levels of the 12 major structural genes in the isoflavone biosynthetic pathway. For each gene, the transcript level for six selected mutant lines and three wild-type cultivars analyzed in this study are displayed as a heatmap based on the normalized *C*_t_ values.

We selected six mutant lines that showed altered TIC for analysis of the expression patterns of isoflavone biosynthetic genes. The genes *CHI1A*, *IFS1*, and *IFS2* showed differences in stages and expression patterns depending on the individual or the wild-type cultivar. The genes *IFS1* and *IFS2* encode proteins that differ by 14 amino acids. The IFS1 and IFS2 proteins convert the flavanones naringenin and liquiritigenin to the corresponding isoflavones genistein and daidzein, respectively [16]. The expression patterns of the *IFS* gene family were consistent with isoflavonoid accumulation in the seed, which indicated there is a close relationship between expression of these genes and metabolite accumulation in the seed [9]. In this study, the *IFS2* relative expression level was significantly higher in mutant lines that exhibited increased isoflavone accumulation compared with lines that showed decreased isoflavone content in the seed. Also, the *MaT7* gene showed consistently higher expression levels at stage 1 in the three IIC lines and may be the predominant contributor to isoflavone biosynthesis in the seeds, while the *MaT3* gene only increased in DP-084 during stage 2. Previous studies have shown the diverse subcellular localization of *MaTs*, such as the cytosol [43], the ER [44], and the nucleus and cytoplasm [14]. In addition, the soybean genome contains a number of *MaT* homologues because of the genome’s paleopolyploid nature [45]. The primary subcellular localization of the protein encoded by *MaT1* in the ER and the protein encoded by *MaT3* in the cytosol might reflect their functions in malonylation following the synthesis of isoflavone glucosides. Similar to other flavonoids, isoflavone glucosides are considered to be synthesized on the cytosolic side of the ER and are subsequently modified by *MaTs* [46]. We found that expression patterns of the major isoflavone structural genes in selected mutants were dependent on the seed developmental stages and thus were also cultivar-specific. So, in order to more clearly define the gene expression patterns, we displayed these relationships in the phenylalanine pathway (Figure 4).

**Figure 5:**
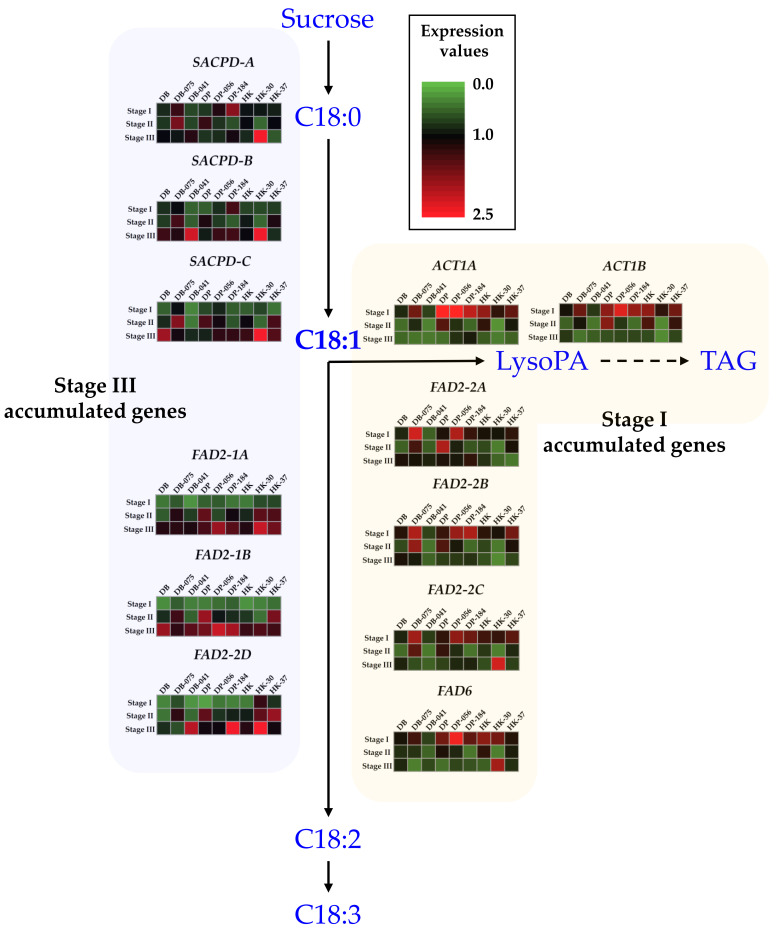
Transcript levels of the 12 major structural genes in the fatty acid biosynthetic pathway. For each gene, the transcript level for six selected mutant lines and three wild-type cultivars analyzed in this study are displayed as a heatmap based on the normalized *C*_t_ values.

The expression level of 12 major structural genes in fatty acid biosynthesis was analyzed in nine selected lines, comprising three IOC mutant lines (DB-075, DP-056, and HK-30), three DOC mutant lines (DB-041, DP-184, and HK-37), and the corresponding cultivars (DB, DP, and HK) at seed developmental stages 1 to 3. In the study, the 12 genes were classifiable into two groups: (i) ES I: highly accumulated in stage 1, consisting of *ACT1A*, *ACT1B*, *FAD2-2A*, *FAD2-2B*, *FAD2-2C*, and *FAD6*; and (ii) ES III: highly accumulated in stage 3, comprising *SACPD-A*, *SACPD-B*, *SACPD-C*, *FAD2-1A*, *FAD2-1B*, and *FAD2-2D* (Figure 5). Previously, we developed mutant soybean populations by gamma irradiation of the cultivars ‘Danbaek’ and ‘Daepung’ and evaluated the linolenic acid content of the seed in 78 and 154 M_9_ mutant progenies. The selected mutant line showed 33.9%–67.7% higher linolenic acid content compared with that of the wild-type cultivars, and increased expression levels of the fatty acid desaturation enzyme (FAD) gene during seed development. In addition, the linolenic acid content was associated with a significant increase in the expression levels of *FAD3C* and *FAD3D* in the ER [47]. According to soybean RNA-seq data, FAD gene expression patterns vary depending on the tissue and developmental stage [48]. In the present study, the microsomal omega-6 desaturase genes *FAD2-1A* and *FAD2-1B* showed increased expression levels among the selected DB-, DP-, and HK-mutants during seed development compared with those of the wild-type cultivars. Similarly, Robert et al [49] reported the gene expression of eight FAD genes in three seed development stages. Among genes, *FAD2-1A* and *FAD2-1B* increased their expression from 22 DAF to 28 DAF stages. However, it decreased at the 35 DAF stages. In addition, the two microsomal FAD2-1 desaturases *FAD2-1A* and *FAD2-1B* were mainly expressed in developing seeds [23]. Thus, *FAD2-1A* and *FAD2-1B* are considered to play an important role in controlling the oleic acid level in soybean seed development. The seed-specific expression of *FAD2-1* and *FAD2-2* plays a role in desaturation of 18:1 in soybean seeds [20]. The majority of acyl carrier proteins and fatty acid elongases were expressed at a higher level during the early maturity stage (stage 2) and subsequently the expression level declined at stage 3. Similarly, soybean also reported that *FAD* expression patterns varied depending on the tissue and developmental stage [49] and in Arabidopsis, the expression level of fatty acid-associated genes is elevated during seed maturation [50].

## 4. Materials and Methods

### 4.1. Plant Materials

**Figure 6:**
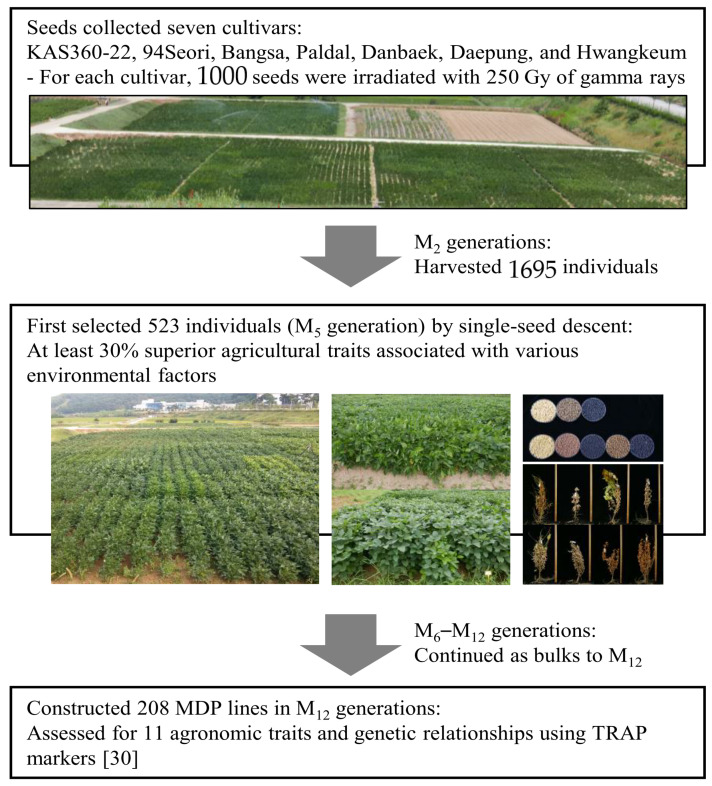
Schematic representation of the development of the 208 MDP lines. In total, 1000 irradiated seeds of each of seven soybean cultivars were sown in a research field of KAERI. Seeds from the 1695 M_2_ plants were harvested and first selected for 523 mutant lines in the M_5_ generation based on agricultural traits such as grain yield, growth type, and climate adaptability. In a second selection phase in the M_12_ generation, 208 genetically fixed mutants were selected to eliminate redundant phenotypes from the 523 mutant lines (additional information is described in [30]).

Soybean seeds were irradiated with gamma-rays generated using a ^60^Co gamma-irradiator (150 TBq capacity; ACEL, Ottawa, ON, Canada) at the Korea Atomic Energy Research Institute (KAERI). The irradiated seeds and control seeds were sown at the Breeding Research Farm at the KAERI. Seeds from the M_1_ plants were harvested individually and carried forward to the M_2_ generation. Genetically fixed mutant lines (M_12_ generation) were selected for desirable agronomic traits, such as grain yield, growth type, climate adaptability, and morphological phenotypes (Figure 6). For analysis of isoflavones and fatty acids, 208 soybean MDP lines, consisting of one landrace (KAS360-22) and six representative Korean soybean cultivars (‘94Seori’, ‘Bangsa’ [BS], ‘Paldal’ [PD], ‘Danbaek’ [DB], ‘Daepung’ [DP], and ‘Hwangkeum’ [HK]), were cultivated from 2008 [30]. Ultimately, we selected 15 MDP lines for gene expression analysis: six isoflavone-altered lines, comprising two DB-mutants (DB-088 and DB-064), two DP-mutants (DP-084 and DP-093), and two HK-mutants (HK-17 and HK25-165); six fatty acid-altered lines, comprising two DB-mutants (DB-075 and DB-041), two DP-mutants (DP-056 and DP-184), and two HK-mutants (HK-30 and HK-37); and the corresponding wild-type cultivars (DB, DP, and HK).

### 4.2. Isoflavone Extraction and Quantification

Lyophilized whole soybean seeds were finely ground with a mortar. Each ground sample (7 mg) was immersed in 1 mL of 58% (*v*/*v*) aqueous acetonitrile. The mixture was sonicated for 30 min and centrifuged at 13,000 rpm for 5 min, after which the supernatant was retained. The pellet was resuspended in an equal volume of solvent, and both retained supernatants were combined and diluted with distilled water. The extract volume was adjusted to 4 mL for each extraction from a 7-mg freeze-dried seed sample. The diluted extracts were filtered through a 0.45-μm syringe filter (Futecs Co., Ltd., Daejeon, Korea) and used for reversed-phase high performance liquid chromatography (HPLC) analysis.

Extracts were analyzed using reversed-phase HPLC (Waters 2695 Alliance HPLC; Waters Inc., Milford, MA, USA) with an octadecylsilane column (Prontosil 120–5-C18-ace-EPS 5.0 μm (250 × 4.6 mm; Bischoff, Leonberg, Germany). The flow rate of the mobile phase was 1.0 mL/min and the sample injection volume was 5 μL. The mobile phase was a combination of (A) water with 0.1% formic acid and (B) acetonitrile with 0.1% formic acid. Gradient elution was performed by adding 15% of solvent B at the initial running time and increasing the concentration to 34% over 60 min. Peaks were monitored at 254 nm using a Waters 996 photodiode array detector (Waters Inc.). Twelve isoflavone standards were purchased from Sigma-Aldrich (St. Louis, MO, USA) and used for quantification of the isoflavones from soybean seeds in the HPLC analysis.

### 4.3. Fatty Acid Analysis

For gas chromatography–mass spectrometry (GC-MS) analysis, fatty acids were extracted as described by Ryu et al. [51] with the following modifications. A powdered freeze-dried seed sample (0.5 g) was extracted in 1 mL *n*-hexane for 4 h, then 0.1 mL of 2 N potassium hydroxide in methanol was added. After centrifugation for 5 min at 3000× *g*, the collected supernatant was filtered using a 0.45-μm syringe filter. The fatty acid composition was analyzed using a GC-MS (Plus-2010, Shimadzu, Japan) instrument equipped with a HP-88 capillary column (J&W Scientific, Folsom, CA, USA, 60 m × 0.25 mm × 0.25 m) under the following conditions: ionization voltage, 70 eV; mass scan range, 50–450 mass units; injector temperature, 230 °C; detector temperature, 230 °C; injection volume, 1 L; split ratio, 1:30; carrier gas, helium; and flow rate, 1.7 mL/min. The column temperature program specified an isothermal temperature of 40 °C for 5 min increasing to 180 °C at the rate of 5 °C/min, then a subsequent increase to 28 °C at the rate of 1 °C/min. We identified the substances present in the extracts in accordance with their retention time and with reference to a mass spectral database (NIST 62 Library).

### 4.4. RNA Isolation and cDNA Synthesis

Immature seeds from the 15 selected MDP lines were collected at stage 1 (length 4 to <7 mm; R5e, DAF20), stage 2 (7–10 mm; R5L, DAF30), and stage 3 (11–14 mm; R6, DAF40) in accordance with a previous report [13] with some modifications (Supplementary Figure S1). Total RNA was isolated from seeds with TRIzol Reagent in accordance with the manufacturer’s protocol (Invitrogen, Carlsbad, CA, USA). The RNA concentration and quality were measured using a NanoDrop ND-1000 spectrophotometer (Thermo Fisher Scientific, Waltham, MA, USA) prior to DNase digestion. For each sample, 15 μg total RNA was digested in a volume of 20 μL using the Invitrogen DNA-free Kit (Life Technologies, Grand Island, NY, USA) to remove genomic DNA contamination following the manufacturer’s instructions. After DNase I digestion, the RNA concentration was determined using a NanoDrop ND-1000 spectrophotometer. First-strand cDNA synthesis was performed using 1 μg DNase-treated total RNA in a 20-μL reaction using the SuperScript III First-Strand Synthesis SuperMix kit (Invitrogen, Carlsbad, CA, USA) following the manufacturer’s instructions.

### 4.5. Quantitative Real-Time PCR

Transcript levels of selected genes involved in the phenylpropanoid and fatty acid pathway were quantified by quantitative real-time PCR (qRT-PCR) analysis. Relative expression level was calculated using the 2^−ΔΔ*C*t^ comparative threshold method [52]. Primer specificity was confirmed by blasting each primer sequence against soybean genome sequences lodged in the Phytozome database (http://www.phytozome.net/, last accessed on 19 May 2021) using the BLASTN algorithm. The qRT-PCR reactions were performed in 96-well plates using the CFX96 Real-Time PCR Detection System (Bio-Rad, Hercules, CA, USA). The iTaq Universal SYBR Green Supermix (Bio-Rad) was used for real-time cDNA quantification. A 10 pmol primer concentration and 3 μL of prepared cDNA were used in a final volume of 20 μL per reaction. The PCR protocol was as follows: 95 °C for 10 min, followed by 40 cycles of 95 °C for 15 s, 50 °C for 15 s, and 72 °C for 30 s. The results were normalized to the constitutive expression level of *ELF1B*, which was selected as an internal reference gene owing to its expression stability. Gene-specific primers used for qRT-PCR analyses are listed in Supplementary Tables S3 and S4.

## 5. Conclusions

In the present study, we analyzed the metabolic properties, including the isoflavones and five fatty acid contents, of 208 MDP lines. The genetically fixed mutant lines that showed significantly increased or decreased isoflavone and fatty acid contents were selected from the DB-, DP-, and HK- mutant population. The lines were selected to analyze the differential expression of isoflavones and fatty acid biosynthetic genes at three seed developmental stages. Isoflavone biosynthetic genes, including *CHI1A*, *IFS1*, and *IFS2*, showed differences in stages and expression patterns depending on the individual or wild-type cultivar, whereas *MaT7* showed consistently higher expression levels in seeds at stage 1. The fatty acid biosynthetic genes were classifiable into two groups based on the developmental stages of the seeds. Our results can serve as a foundation for future functional analysis of the regulatory genes involved in the isoflavone and fatty acid biosynthetic pathways.

## Figures and Tables

**Table 1:** Variation of isoflavone contents (mg/g dry weight) in seeds of 208 soybean MDP lines.

Lines	Values	TIC ^x^
KAS360-22 (N = 2)	Minimum	0.88
	Maximum	2.02
	Mean	1.45
	SD ^y^	0.81
	CV (%) ^z^	55.59
94seori (N = 5)	Minimum	1.27
	Maximum	2.42
	Mean	1.82
	SD	0.44
	CV (%)	24.22
Bangsa (N = 6)	Minimum	1.05
	Maximum	2.26
	Mean	1.36
	SD	0.47
	CV (%)	34.48
Paldal (N = 16)	Minimum	1.14
	Maximum	4.07
	Mean	2.45
	SD	0.91
	CV (%)	37.31
Danbaek (N = 65)	Minimum	1.03
	Maximum	7.12
	Mean	3.43
	SD	1.46
	CV (%)	42.55
Daepung (N = 61)	Minimum	1.59
	Maximum	5.04
	Mean	3.46
	SD	1.06
	CV (%)	30.75
Hwangkeum (N = 53)	Minimum	1.08
	Maximum	3.21
	Mean	2.05
	SD	0.53
	CV (%)	26.02

^z^ CV: Coefficient of variance, ^y^ SD: Standard deviation, ^x^ TIC: Total isoflavone content.

**Table 2:** Variation of fatty acid contents (%) in seeds of 208 soybean MDP lines.

Lines	Values	PA ^x^	SA ^w^	OA ^v^	LA ^u^	ALA ^t^
KAS360-22 (N = 2)	Minimum	15.37	3.66	2.25	50.59	5.28
	Maximum	18.89	22.99	12.13	59.00	9.84
	Mean	17.13	13.33	7.19	54.80	7.56
	SD ^y^	2.49	13.67	6.99	5.95	3.22
	CV (%) ^z^	14.53	102.58	97.17	10.85	42.65
94seori (N = 5)	Minimum	15.26	2.22	9.55	15.26	6.91
	Maximum	17.92	3.83	14.98	63.90	9.90
	Mean	16.80	3.05	11.45	60.27	8.43
	SD	1.18	0.65	2.29	3.23	1.13
	CV (%)	7.04	21.33	20.00	5.37	13.43
Bangsa (N = 6)	Minimum	17.57	2.81	4.11	58.83	9.14
	Maximum	19.03	3.39	9.16	64.96	11.58
	Mean	18.26	3.11	6.48	61.53	10.63
	SD	0.57	0.23	2.02	2.15	0.88
	CV (%)	3.15	7.46	31.13	3.53	8.32
Paldal (N = 16)	Minimum	14.51	2.34	4.30	55.51	7.71
	Maximum	17.94	3.72	16.95	66.16	11.02
	Mean	15.76	3.02	10.29	61.87	9.06
	SD	0.87	0.41	2.97	2.50	0.98
	CV (%)	5.52	13.70	28.87	4.05	10.81
Danbaek (N = 65)	Minimum	12.46	0.99	0.38	57.53	5.97
	Maximum	20.64	4.12	15.43	68.69	14.00
	Mean	16.13	2.60	9.34	62.79	9.14
	SD	1.77	0.63	2.79	2.01	1.28
	CV (%)	10.95	24.23	29.87	3.20	14.05
Daepung (N = 61)	Minimum	13.05	0.00	1.54	56.51	5.62
	Maximum	20.28	3.23	19.83	73.40	12.10
	Mean	16.24	1.48	10.03	63.89	8.35
	SD	1.71	1.04	4.55	4.03	1.45
	CV (%)	10.52	70.25	45.37	6.31	17.40
Hwangkeum (N = 53)	Minimum	12.42	0.00	0.41	53.11	1.00
	Maximum	21.00	7.49	24.66	74.70	13.28
	Mean	16.19	1.20	15.05	61.40	6.15
	SD	1.70	1.49	6.03	4.45	2.12
	CV (%)	10.50	123.96	40.06	7.25	34.38

^z^ CV: Coefficient of variance, ^y^ SD: Standard deviation, ^x^ PA: Palmitic acid, ^w^ SA: Stearic acid, ^v^ OA: Oleic acid, ^u^ LA: Linoleic acid, ^t^ ALA: a-Linolenic acid.

## Supplementary Materials

The following are available online at https://www.mdpi.com/article/10.3390/plants10061037/s1, Figure S1: Soybean seed developmental stages. Stage 1, length 4 to <7 mm; stage 2, length 7–10 mm; and stage 3, length 11–14 mm, Table S1: Total isoflavone content in the seeds of 208 soybean MDP lines, Table S2: Fatty acid content in the seeds of 208 soybean MDP lines, Table S3: Primer pairs used for quantitative real-time PCR analysis (isoflavone biosynthesis genes), Table S4: Primer pairs used for quantitative real-time PCR analysis (fatty acid biosynthesis genes).
