# Supplementary material for: Differential Gene Expression Associated with Altered Isoflavone and Fatty Acid Contents in Soybean Mutant Diversity Pool
Source: Plants (Basel). 2021 May 21;10(6):1037. doi: 10.3390/plants10061037 (PMC8224098; doi:10.3390/plants10061037)
Supplement: Supplementary file 1 [file plants-10-01037-s001.zip › Supplementary Table S1_total isoflavone content.pdf]

plants

MDPI

Table S. Total isoflavone content in the seeds of 208 soybean MDP lines.

No.	Lines	Total isoflavone content	No.	Lines	Total isoflavone content
1	94seori	1.97	105	DP-053	3.69
2	Joseangseori	1.27	106	DP-054	4.63
3	J-S	2.42	107	DP-055	5.25
4	J-D01	1.53	108	DP-056	5.78
5	J-D02	1.89	109	DP-057	4.11
6	KAS360-22	0.88	110	DP-058	4.39
7	KAS360-22-W	2.02	111	DP-059	5.55
8	BangSa	2.26	112	DP-060	3.16
9	BS-25	1.51	113	DP-061	5.56
10	BS-63	1.07	114	DP-062	4.38
11	BS-73	1.14	115	DP-079	6.98
12	BS-74	1.15	116	DP-080	6.68
13	BS-84	1.05	117	DP-081	5.8
14	PalDal	2.31	118	DP-082	4.24
15	P-D01	2.35	119	DP-083	5
16	P-D02	1.52	120	DP-084	7.08
17	P-D03	2.4	121	DP-085	5.85
18	P-D04	1.81	122	DP-086	5.9
19	P-D05	1.81	123	DP-087	3.83
20	P-D06	1.88	124	DP-088	3.47
21	P-D07	1.14	125	DP-089	5.66
22	P-I01	1.27	126	DP-090	6.55
23	P-I02	2.27	127	DP-091	5.33
24	P-I03	2.93	128	DP-092	4.14
25	P-I04	3.47	129	DP-093	2.51
26	P-I05	3.6	130	DP-094	3.05
27	P-I06	2.46	131	DP-095	3.32
28	P-I07	3.91	132	DP-097	4.24
29	P-I08-W	4.07	133	DP-098	2.85
30	DanBaek	1.03	134	DP-104	4.08
31	DB-003	1.29	135	DP-106	3.54
32	DB-004	2.34	136	DP-107	1.47
33	DB-005	1.85	137	DP-111	4.56
34	DB-006	1.61	138	DP-114	5.04
35	DB-007	2.48	139	DP-117	2.89
36	DB-008	2.27	140	DP-120	3.94
37	DB-009	4.59	141	DP-121	4.26

38	DB-010	1.9	142	DP-127	4.01
39	DB-016	2.69	143	DP-129	2.85
40	DB-019	3.24	144	DP-131	4.35
41	DB-024	3	145	DP-132	2.14
42	DB-026	2.51	146	DP-140	3.23
43	DB-027	3.62	147	DP-152	3.29
44	DB-029	3.47	148	DP-172	3.27
45	DB-030	4.01	149	DP-178	3.11
46	DB-031	4	150	DP-179	2.83
47	DB-033	3.58	151	DP-183	3.98
48	DB-034	3.71	152	DP-184	3.82
49	DB-035	6.63	153	DP-190	2.19
50	DB-036	5.11	154	DP-192	4.16
51	DB-037	2.78	155	DP-200	3.22
52	DB-038	4.11	156	HwangKeum	1.85
53	DB-039	4.88	157	HK-1	2.37
54	DB-040	2.73	158	HK-2	2.88
55	DB-041	4.73	159	HK-3	2.65
56	DB-044	3.51	160	HK-4	2.24
57	DB-045	3.45	161	HK-5	2
58	DB-046	4.6	162	HK-6	2.68
59	DB-049	6.76	163	HK-7	2.57
60	DB-050	4.2	164	HK-8	3.13
61	DB-051	6.34	165	HK-9	2.24
62	DB-054	3.99	166	HK-10	2.56
63	DB-056	2.7	167	HK-11	2.31
64	DB-058	3.5	168	HK-12	2.64
65	DB-059	2.15	169	HK-13	2.4
66	DB-060	1.48	170	HK-14	2.36
67	DB-061	2.54	171	HK-15	1.92
68	DB-062	2.31	172	HK-16	1.86
69	DB-063	2.46	173	HK-17	3.21
70	DB-064	1.34	174	HK-18	2.49
71	DB-065	2.86	175	HK-19	1.95
72	DB-066	2.23	176	HK-20	2.11
73	DB-067	3.85	177	HK-21	1.52
74	DB-068	2.95	178	HK-22	1.37
75	DB-069	1.2	179	HK-23	1.61
76	DB-072	4.2	180	HK-24	1.61
77	DB-073	1.8	181	HK-25	1.85
78	DB-074	3.67	182	HK-27	1.58
79	DB-075	2.36	183	HK-28	1.36

80	DB-076	2.77	184	HK-29	1.37
81	DB-077	2.23	185	HK-30	1.64
82	DB-078	4.48	186	HK-31	2.07
83	DB-079	2.42	187	HK-32	2.11
84	DB-080	2.6	188	HK-33	1.93
85	DB-083	4.34	189	HK-34	2.01
86	DB-085	3.21	190	HK-35	2.35
87	DB-086	7.11	191	HK-36	2.48
88	DB-087	4.2	192	HK-37	1.4
89	DB-088	7.12	193	HK-38	3.06
90	DB-089	6.71	194	HK-39	1.52
91	DB-090	4.01	195	HK-40	2.22
92	DB-091	3.19	196	HK-41	2.07
93	DB-092	4.45	197	HK-42	2.15
94	DB-093	3.39	198	HK-43	3.14
95	DaePung	4.95	199	HK-44	2.19
96	DP-009	1.59	200	HK-45	2.16
97	DP-012	3.69	201	HK-46	1.47
98	DP-027	2.97	202	HK-47	1.6
99	DP-028	3.96	203	HK-48	1.45
100	DP-029	3.8	204	HK-49	1.56
101	DP-046	2.62	205	HK-50	1.3
102	DP-048	3.26	206	HK-60	1.87
103	DP-051	5.04	207	HK25-78	1.08
104	DP-052	2.71	208	HK25-165	1.18
