# Supplementary material for: Differential Gene Expression Associated with Altered Isoflavone and Fatty Acid Contents in Soybean Mutant Diversity Pool
Source: Plants (Basel). 2021 May 21;10(6):1037. doi: 10.3390/plants10061037 (PMC8224098; doi:10.3390/plants10061037)
Supplement: Supplementary file 1 [file plants-10-01037-s001.zip › Supplementary Table S2_fatty acid content.pdf]

plants

Table S2. Fatty acid content in the seeds of 208 soybean MDP lines.

NO.	Lines	16:0	18:0	18:1	18:2	18:3
1	94seori	15.26	3.83	14.98	56.03	9.9
2	Joseangseori	17.92	2.22	10.59	61.03	8.24
3	J-S	17.88	3.11	9.67	62.43	6.91
4	J-D01	15.93	2.61	9.55	63.9	8.01
5	J-D02	17.01	3.5	12.45	57.94	9.1
6	KAS360-22	18.89	22.99	2.25	50.59	5.28
7	KAS360-22-W	15.37	3.66	12.13	59	9.84
8	BangSa	17.94	3.39	4.57	64.96	9.14
9	BS-25	19.03	2.81	4.11	62.47	11.58
10	BS-63	18.67	3.08	5.99	62.18	10.08
11	BS-73	17.57	3.23	9.16	58.83	11.21
12	BS-74	18.54	2.86	6.66	60.95	10.99
13	BS-84	17.78	3.26	8.38	59.81	10.77
14	PalDal	16.19	3.58	16.95	55.51	7.77
15	P-D01	16.25	2.96	10.62	61.16	9.01
16	P-D02	15.19	3.07	12.42	60.82	8.5
17	P-D03	14.51	3.09	11.8	62.24	8.36
18	P-D04	16.35	2.77	8.64	62.98	9.26
19	P-D05	15.18	2.95	12.18	60.81	8.88
20	P-D06	15.1	2.69	12.82	60.01	9.38
21	P-D07	15.3	2.52	8.63	63.6	9.95
22	P-I01	16.67	2.37	8.4	63.59	8.97
23	P-I02	16.18	3.37	9.28	62.35	8.82
24	P-I03	15.21	3.24	6.94	63.59	11.02
25	P-I04	15.17	3.51	10.74	60.5	10.08
26	P-I05	14.73	3.19	13.25	60.94	7.89
27	P-I06	17.94	2.97	4.3	66.16	8.63
28	P-I07	15.97	3.72	9.39	60.24	10.68
29	P-I08-W	16.21	2.34	8.25	65.49	7.71
30	DanBaek	17.97	1.72	5.83	64.84	9.64
31	DB-003	17.91	2.97	5.52	62.8	10.8
32	DB-004	18.69	1.98	6.68	66.68	5.97
33	DB-005	16.6	2.25	7.64	64.39	9.12
34	DB-006	16.1	3.99	10.24	62.46	7.21
35	DB-007	16.96	3.17	11.33	60.8	7.74
36	DB-008	15.9	2.44	9.58	63.66	8.42
37	DB-009	16.84	3.53	8.43	60.27	10.93
38	DB-010	16.81	3.96	13.28	58.42	7.53
39	DB-016	18.01	2.88	9.49	60.99	8.63
40	DB-019	15.94	2.9	9.88	62.86	8.42

41	DB-024	17.5	3.2	10.28	60.94	8.08
42	DB-026	15.06	1.48	12.64	62.82	8
43	DB-027	13.44	2.43	9.94	63.25	10.94
44	DB-029	13.58	2.87	12.01	62.14	9.4
45	DB-030	14.86	2.84	11.21	61.88	9.21
46	DB-031	14.75	2.55	9.64	63.04	10.02
47	DB-033	14.17	2.27	12.37	61.7	9.49
48	DB-034	14.45	3.09	11.44	61.27	9.75
49	DB-035	14.57	3.51	10.39	61.31	10.22
50	DB-036	13.23	2.72	13.08	62.47	8.5
51	DB-037	15.4	3.14	10.47	62.08	8.91
52	DB-038	14.96	3.4	9.94	62.66	9.04
53	DB-039	15.37	2.28	11.43	61.22	9.7
54	DB-040	14.55	2.45	9.27	63.45	10.28
55	DB-041	19.38	1.81	0.38	68.69	9.74
56	DB-044	18.88	2.8	3.58	65.99	8.75
57	DB-045	19.03	2.6	5.19	64.8	8.38
58	DB-046	17.36	3.11	6.7	65.25	7.58
59	DB-049	17.13	3.19	6.75	64.51	8.42
60	DB-050	16.82	3.07	7.51	64.18	8.42
61	DB-051	17.03	2.62	8.63	62.13	9.59
62	DB-054	17.75	3.56	4.99	63.96	9.74
63	DB-056	16.15	2.75	8.82	64.32	7.96
64	DB-058	16.21	4.12	8.51	63.92	7.24
65	DB-059	19.59	2.58	7.52	60.82	9.49
66	DB-060	20.64	2.11	6.89	62.16	8.2
67	DB-061	17.59	0.99	8.47	65.52	7.43
68	DB-062	16.82	2.15	8.25	62.92	9.86
69	DB-063	17.57	1.46	6.07	66.18	8.72
70	DB-064	17.3	1.83	5.52	65.74	9.61
71	DB-065	18.34	2.11	7.87	64.17	7.51
72	DB-066	15.44	2.47	8.63	62.91	10.55
73	DB-067	16.32	2.56	8.27	63.44	9.41
74	DB-068	16.11	1.88	4.83	66.35	10.83
75	DB-069	16	3.42	9.76	62.83	7.99
76	DB-072	15.4	2.37	10.95	62.02	9.26
77	DB-073	16.25	2.52	11.43	61.2	8.6
78	DB-074	13.87	2.61	12.42	61.45	9.65
79	DB-075	15.69	2.55	15.43	57.53	8.8
80	DB-076	15.14	2.97	14.06	59.77	8.06
81	DB-077	15.87	2.69	11.01	60.01	10.42
82	DB-078	15.68	2.34	10.94	62.6	8.44

83	DB-079	16.12	2.58	10.56	62.23	8.51
84	DB-080	16.29	2.83	11.89	60.75	8.24
85	DB-083	14.7	2.28	8.88	64.17	9.97
86	DB-085	14.79	2.87	13.54	59.9	8.9
87	DB-086	13.41	2.55	13.45	60.88	9.71
88	DB-087	13.01	2.19	11.87	61.5	11.43
89	DB-088	12.46	1.47	10.53	61.54	14
90	DB-089	13.62	2.23	7.66	64.3	12.19
91	DB-090	14.14	1.61	12.04	62.77	9.44
92	DB-091	14.66	1.93	10.99	64.17	8.25
93	DB-092	17.75	2.51	6.73	63.28	9.73
94	DB-093	18.34	2.38	7.79	62.22	9.27
95	DaePung	17.37	3.04	6.05	66.01	7.53
96	DP-009	19.99	1.01	2.98	63.92	12.1
97	DP-012	18.25	3.23	10.75	59.82	7.95
98	DP-027	18.75	1.89	7.07	66.33	5.96
99	DP-028	17.63	1.16	7.81	67.04	6.36
100	DP-029	15.91	2.96	10.53	62.26	8.34
101	DP-046	15.35	1.03	14.64	62.02	6.96
102	DP-048	15.68	2.16	11.51	63.02	7.63
103	DP-051	16.33	3.15	10.43	62.41	7.68
104	DP-052	14.7	2.13	17.72	57.28	8.17
105	DP-053	15.35	3.02	13.54	59.34	8.75
106	DP-054	15.6	1.89	12.01	61.74	8.76
107	DP-055	14.61	1.73	19.83	56.51	7.32
108	DP-056	14.08	2.83	19.68	56.94	6.47
109	DP-057	14.62	2.38	17.29	58.52	7.19
110	DP-058	15.28	1.43	17.9	58.82	6.57
111	DP-059	14.41	2.23	18.56	58.5	6.3
112	DP-060	15.8	2.81	9.82	63.17	8.4
113	DP-061	15.82	1.71	15.31	59.92	7.24
114	DP-062	14.99	2.22	16.14	58.15	8.5
115	DP-079	13.05	2.26	13.73	60.89	10.07
116	DP-080	13.65	2.19	12.35	62.48	9.33
117	DP-081	14.38	2.45	12.17	61.9	9.1
118	DP-082	13.24	2.16	14.93	61.4	8.27
119	DP-083	13.45	1.87	11.57	63.13	9.98
120	DP-084	14.98	2.28	9.15	63.74	9.85
121	DP-085	20.28	0.5	3.22	67.04	8.96
122	DP-086	18.39	0	2.99	67.77	10.85
123	DP-087	19.68	0.51	6.03	64.19	9.59
124	DP-088	18.4	1.82	13.55	58.42	7.81

125	DP-089	16.72	2.23	13.36	60.19	7.5
126	DP-090	17.65	0	1.54	72.74	8.07
127	DP-091	15.73	0	6.77	68.82	8.68
128	DP-092	17.19	2.7	2.83	66.63	10.65
129	DP-093	17.98	0	4.39	71.44	6.19
130	DP-094	15.64	1.64	7.65	67.47	7.6
131	DP-095	16.71	0	8.86	67.21	7.22
132	DP-097	16.02	0	6.71	68.02	9.25
133	DP-098	15.01	0.72	14.72	62.25	7.3
134	DP-104	17.87	0.5	6.24	66.65	8.74
135	DP-106	16.22	1.26	8.13	64.02	10.37
136	DP-107	16.75	0	8.06	65.04	10.15
137	DP-111	15.29	0.8	9.61	64.53	9.77
138	DP-114	15.98	1.1	7.75	65.86	9.31
139	DP-117	15.91	1.93	10.21	62.46	9.49
140	DP-120	16.68	1.09	12.88	58.86	10.49
141	DP-121	14.95	2.57	12.19	60.53	9.76
142	DP-127	16.15	2.61	7.74	65.44	8.06
143	DP-129	14.68	1.94	14.12	62	7.26
144	DP-131	15.94	1.26	10.35	61.86	10.59
145	DP-132	15.48	1.75	11.38	63.79	7.6
146	DP-140	16.38	1.65	9.28	62.87	9.82
147	DP-152	13.96	1.76	11.86	64.82	7.6
148	DP-172	15.73	0	6.72	67.62	9.93
149	DP-178	18.81	0	5.19	66.99	9.01
150	DP-179	19.73	0	4.51	69.24	6.52
151	DP-183	18.32	0	7.27	66.88	7.53
152	DP-184	18.26	0	3.81	71.42	6.51
153	DP-190	16.63	0.1	4.25	73.4	5.62
154	DP-192	15.84	2.73	7.59	64.89	8.95
155	DP-200	16.42	0	6.71	70.95	5.92
156	HwangKeum	15.96	2.3	18.52	56.63	6.59
157	HK-1	18.14	1.14	8.44	63.95	8.33
158	HK-2	17.61	1.78	10.84	61.44	8.33
159	HK-3	15.62	2.05	19.79	57.34	5.2
160	HK-4	15.72	1.97	16.61	59.63	6.07
161	HK-5	18.48	0.6	12.45	62.24	6.23
162	HK-6	18.26	2.8	8.63	60.71	9.6
163	HK-7	16.12	0.96	20.58	55.27	7.07
164	HK-8	15.36	0.33	12.79	61.66	9.86
165	HK-9	14.74	0	21.87	60.43	2.96
166	HK-10	16.09	0	20.23	58.02	5.66

167	HK-11	15.38	0.71	16.28	59.18	8.45
168	HK-12	16.26	2.08	17.01	57.63	7.02
169	HK-13	14.09	1.28	21.28	57.03	6.32
170	HK-14	14.51	2.25	20.35	57.98	4.91
171	HK-15	14.77	1.5	20.1	58.83	4.8
172	HK-16	14.99	0.3	17.19	60.65	6.87
173	HK-17	16.44	0.73	14.87	60.67	7.29
174	HK-18	15.09	1.19	18.19	59.58	5.95
175	HK-19	14.12	1.32	21.36	56.81	6.39
176	HK-20	13.8	2.5	24.58	53.11	6.01
177	HK-21	16.05	2.55	13.12	61.58	6.7
178	HK-22	20.05	2.43	7.77	60.95	8.8
179	HK-23	19.65	0.25	1.81	69.54	8.75
180	HK-24	17.87	0	14.22	63.41	4.5
181	HK-25	16.82	0	11.14	67.79	4.25
182	HK-27	17.55	1	13.27	62.17	6.01
183	HK-28	15.83	1.3	14.88	61.26	6.73
184	HK-29	14.95	2.63	18.99	57.38	6.05
185	HK-30	14.44	1.21	24.66	55.72	3.97
186	HK-31	17.68	0	13.74	61.72	6.86
187	HK-32	16.79	0	12.33	66.7	4.18
188	HK-33	16.22	0	14.53	63.53	5.72
189	HK-34	16.35	0	14.99	61.08	7.58
190	HK-35	15.49	0	17.93	63.34	3.24
191	HK-36	15.96	0	19.2	61.8	3.04
192	HK-37	18.41	0	0.41	70.91	10.27
193	HK-38	16.47	1.24	15.59	59.39	7.31
194	HK-39	16.14	0	13.6	65.34	4.92
195	HK-40	16.39	0	20.18	62.43	1
196	HK-41	15.72	0	21.93	57.95	4.4
197	HK-42	15.36	0	5.93	65.43	13.28
198	HK-43	13.89	3.96	22.43	53.58	6.14
199	HK-44	15.67	0	10.39	68.53	5.41
200	HK-45	14.35	1.96	22.1	56.44	5.15
201	HK-46	21	0	8.03	64.08	6.89
202	HK-47	13.15	1.23	23.34	57.11	5.17
203	HK-48	12.42	2.19	22.06	57.43	5.9
204	HK-49	18.59	0	11.15	66.73	3.53
205	HK-50	16.65	0.72	2.29	74.7	5.64
206	HK-60	17.19	0	9.05	66.62	7.14
207	HK25-78	16.95	5.84	7.89	66.3	3.02
208	HK25-165	16.37	7.49	6.87	64.6	4.67
