# Supplementary material for: Differential Gene Expression Associated with Altered Isoflavone and Fatty Acid Contents in Soybean Mutant Diversity Pool
Source: Plants (Basel). 2021 May 21;10(6):1037. doi: 10.3390/plants10061037 (PMC8224098; doi:10.3390/plants10061037)
Supplement: Supplementary file 1 [file plants-10-01037-s001.zip › Supplementary Table S3_Primer pairs.pdf]

plants

MDPI

Table S3. Primer pairs used for quantitative real-time PCR analysis (isoflavone biosynthesis genes).

Name	Locus tag	Description	Forward primer	Reverse primer
Gm4CL	*Glyma.* 01g232400	4-coumarate:CoA ligase	AGGCAATGTACGTGGACAAGCT	TCCGAGAGGACAGAGAAGTGGA
GmCHS1	*Glyma.* 08g109400	Chalcone synthase 1	AAGCGCATGTGTGATAAGTCGA	TTGCATCCAACGAAGGTGC
GmCHS7	*Glyma.* 01g228700	Chalcone synthase 7	AACCCACCAAACCGTGTTGAT	CTTGTCACACATGCGCTGAAAT
GmCHI1A	*Glyma.* 20g241500	Chalcone isomerase 1A Type II	GGCGCTGAATACTCAAAGAAGG	AGAGGCACCAGGTGCAAAATT
GmIFS1	*Glyma.* 07g202300	Isoflavone synthase 1	AGAATTCCGTCCCGAGAGGTT	TGCCATTCCTGAAGTAGCCAA
GmIFS2	*Glyma.* 13g173500	Isoflavone synthase 2	AATGTGCCCTGGAGTCAATCTG	GGCGTCACCACCCTTCAATAT
GmF3H	*Glyma.* 02g048400	Flavanone 3-hydroxylase	CCGTTTGTCCATAGCCACTT	GCCTCAAGTTTTGCCTTCTG
GmMT7	*Glyma.* 13g056100	isoflavone-7-0-B-glucoside 6"-O-malonyltransferase	GGCTATTCGATAGGAATAACAAGCC	GTAGTGAAAACAGCAACGGTTCT T
GmMaT1	*Glyma.* 18g268200	malonyl-transferase	ATAGAGGAGCAGAATCAGAGCC	CAATCCACTGTGAACGCAAA
GmMaT3	*Glyma.* 13g056100	malonyl-transferase	TAACAACCACCGCCGAAAC	ACAAATATGCGCCCACGAT
GmUGT1	*Glyma.* 16g175600	glycosyl-transferase	ACATCACGTGCTTCGTTGGA	TGCCAAAGAGCTAACAGGC
GmUGT9	*Glyma.* 09g127200	glycosyl-transferase	AGCGAAAGCAGAAGAAGGG	GACGAGTATTACATGGGGTTAAG C

GmELF1B	*Glyma.* 02g44460	Eukaryotic elongation factor 1 b	GTTGAAAAGCCAGGGGACA	TCTTACCCCTTGAGCGTGG
