# Supplementary material for: Differential Gene Expression Associated with Altered Isoflavone and Fatty Acid Contents in Soybean Mutant Diversity Pool
Source: Plants (Basel). 2021 May 21;10(6):1037. doi: 10.3390/plants10061037 (PMC8224098; doi:10.3390/plants10061037)
Supplement: Supplementary file 1 [file plants-10-01037-s001.zip › Supplementary Table S4_Primer pairs.pdf]

plants

MDPI

Table S4. Primer pairs used for quantitative real-time PCR analysis (fatty acid biosynthesis genes).

Name	Locus tag	Description	Forward primer	Reverse primer
FAD2-1A	*Glyma.*10g278000	omega-6 fatty acid desaturase	GCAAGCCACTACCACCCTTA	ACATAGCAGCCAAACCAACC
FAD2-1B	*Glyma.*20g111000	omega-6 fatty acid desaturase	TCCCAAAACCAAAATCCAAA	TGGGAGCATAAGGGTGGTAG
FAD2-2A	*Glyma.*19g147300	omega-6 fatty acid desaturase	TTGGCCTTGTTCTCCACTCT	TGTAGCGTCCATTGCATGAT
FAD2-2B	*Glyma.*19g147400	omega-6 fatty acid desaturase	TTGTTGGCCTTATCCTCCAC	GACTCTGCCTGGTGGATTGT
FAD2-2C	*Glyma.*03g144500	omega-6 fatty acid desaturase	TGTTCTCCACAATGCCACAT	CACATTGCCTTGACAAATGG
FAD2-2D	*Glyma.*09g111900	omega-6 fatty acid desaturase	AACTCACCAGGGAGGGTTCT	GGGGCCATAAGGGTCATAGT
SACPD-A	*Glyma.*07g207200	stearoyl-acyl carrier protein desaturase	GCAGCTAACCGGACTTTCAG	TTGACGACTCCTTGCCTCTT
SACPD-B	*Glyma.*02g138100	stearoyl-acyl carrier protein desaturase	TGAGAAACGCCATGAGACTG	CCGTCATACATGAGGTGTGC
SACPD-C	*Glyma.*14g121400	stearoyl-acyl carrier protein desaturase	GAACGGTGGAGATTGGAGAA	TGAACTTAACGCCATGATGC
FAD6	*Glyma.*02g203300	omega-6 fatty acid desaturase	CTCCAAGAAAACTGGGGACA	GGCATGGTTTCCTTCAAAAA
ACT1A	*Glyma.* 09g34110	Glycerol-3-phosphate acyltransferase	CTTAAGCAGGGGCACAACAT	CCGGCTACATAGGTCATGCT
ACT1B	*Glyma.* 01g01800	Glycerol-3-phosphate acyltransferase	TAGTCTGGATTGCCCCAAGT	GCACAATATCGCCAAAGGAT
ELF1B	*Glyma.* 02g44460	Eukaryotic elongation factor 1 b	GTTGAAAAGCCAGGGGACA	TCTTACCCCTTGAGCGTGG
