# Supplementary figures and images for: Differential Gene Expression Associated with Altered Isoflavone and Fatty Acid Contents in Soybean Mutant Diversity Pool
Source: Plants (Basel). 2021 May 21;10(6):1037. doi: 10.3390/plants10061037 (PMC8224098; doi:10.3390/plants10061037)

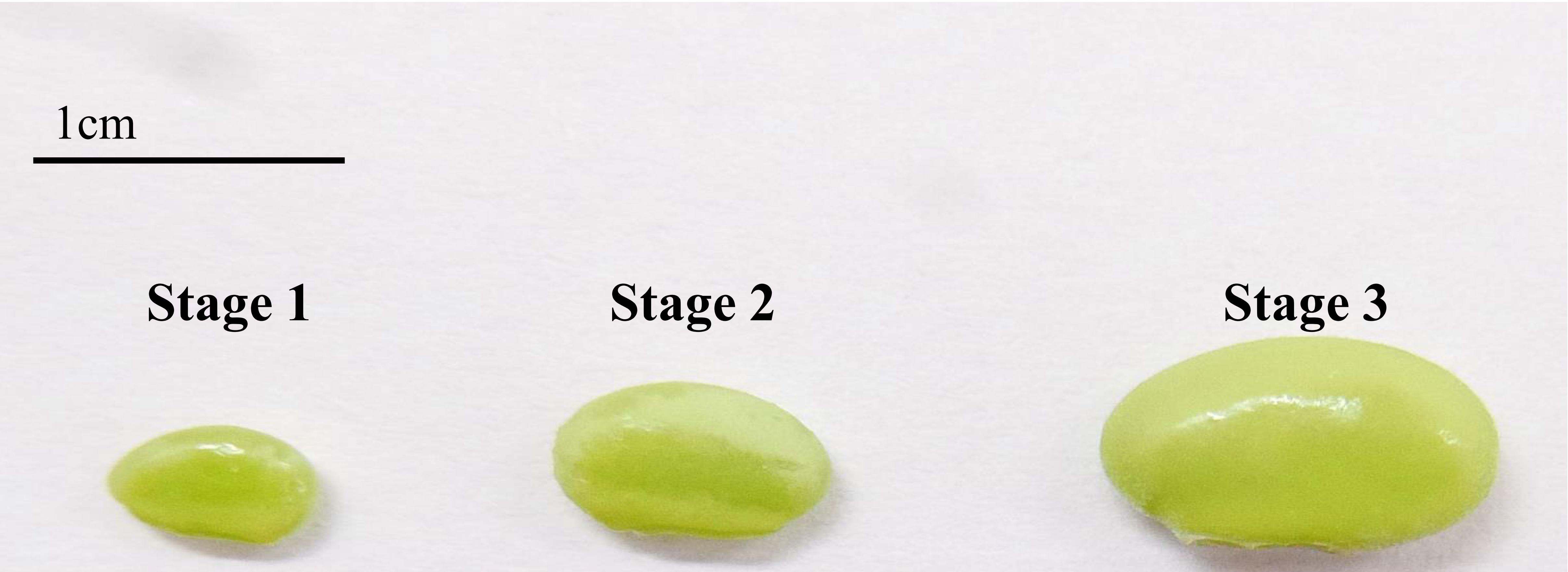

Supplement: Supplementary file 1 [file plants-10-01037-s001.zip › Supplementary Figure S1.tif]
